# Sustaining Health Promotion and Education to Build Resilient Communities: Lessons from Nurses During the COVID-19 Pandemic

**DOI:** 10.3390/ijerph23030366

**Published:** 2026-03-13

**Authors:** Lilian Akorfa Ohene, Merri Iddrisu, Lydia Aziato

**Affiliations:** 1 Department of Public Health Nursing, School of Nursing and Midwifery, College of Health Sciences, University of Ghana, Accra P.O. Box LG 43, Ghana; lohene@ug.edu.gh (L.A.O.); laziato@uhas.edu.gh (L.A.); 2 Department of Adult Health Nursing, School of Nursing and Midwifery, College of Health Sciences, University of Ghana, Accra P.O. Box LG 43, Ghana; 3 Central Administration, University of Health and Allied Sciences, Ho P.O. Box PMB 31, Ghana

**Keywords:** community resilience, COVID-19, Ghana, health education, health promotion, nurses

## Abstract

**Highlights:**

**Public Health Relevance—How does this work relate to a public health issue?**

- Nurses as Frontline Leaders: The study highlights the critical leadership roles of nurses in public health promotion and education to strengthen community resilience and response to public health emergencies.
- Adaptive Strategies: Describing the adaptive strategies of nurses during COVID-19 serves as a model to optimize the health system’s readiness for similar future crises.

**Public Health Significance—Why is this work of significance to public health?**

- Showcasing Systemic Problem: The study illustrates systemic problems that nurses face, such as resource paucity and stigma, and underscores the necessity for complete support systems to have a resilient healthcare domain.
- Inter-professional Teamwork: It underscores the necessity of inter-professional teamwork and a dynamic approach to task setting in disaster scenarios, which can be helpful for health care delivery during calamities.

**Public Health Implications —What are the key implications or messages for practitioners, policy makers and/or researchers in public health?**

- Policy implications: The study concludes that policy points to the need for resource allocation to healthcare workers’ well-being and mental health support to sustain health promotion during and post health crises.
- Community Engagement Approaches: The manuscript argues that community-level outreach and education are essential for addressing misinformation and increasing public health literacy during emergencies.

**Abstract:**

Background: Nurses, the largest segment of the global health workforce, play vital roles in managing disease outbreaks and boosting community resilience during public health emergencies. Purpose: This study explored the experiences of senior nurses in leading health facilities in Ghana during the COVID-19 pandemic. Methods: We employed a qualitative, exploratory, descriptive approach and purposive sampling to recruit 30 senior nurses involved in frontline care during the COVID-19 pandemic. We used telephone interviews to examine how nurses’ roles are changing during public health crises. Results: Nurses navigated challenges related to infection prevention and control, team dynamics and social support, resource limitations, stigma against those affected, and leadership. Some of the nurses drew on their prior experience to navigate the complexities of COVID-19. The significance of inter-professional working and the flexible delegation of tasks is reinforced by the current study, which suggests that professional boundaries became more blurred during the crisis. Optimal responses to outbreaks are influenced by professional preparedness and adaptive learning. Conclusions: Nurses displayed extraordinary resilience and determination, yet faced enormous challenges, including PPE shortages, stigmatization from within their own communities and organizations, and a lack of welfare support. The findings from this analysis are intended to support national and global efforts in pandemic preparedness and healthcare worker assistance, highlighting the essential role nurses play in creating more resilient health systems for future crises.

## 1. Introduction

Health systems worldwide serve as the backbone of public health, especially during crises. The ability to adapt and respond to global health emergencies is crucial for the well-being and survival of entire communities. Nurses are at the heart of these systems, comprising the largest segment of the global health workforce. They serve as indispensable frontline responders in disease outbreaks and other public health emergencies [1,2]. Nurses’ roles extend well beyond direct patient care; they are integral in health education, triage, infection prevention and control, and the implementation of community-based interventions [3]. These multidimensional roles of nurses are particularly vital in low- and middle-income countries (LMICs), such as those in Sub-Saharan Africa, where health systems frequently grapple with limited resources and inadequate infrastructure [4,5].

The COVID-19 pandemic has exerted unprecedented pressure on health systems and the health workforce, testing their resilience and leadership [6,7]. In Sub-Saharan Africa, and Ghana in particular, the pandemic has exacerbated existing challenges, such as inadequate healthcare infrastructure, workforce shortages, and disrupted supply chains, while exposing new vulnerabilities [5]. Despite these difficulties, nurses have remained the cornerstone of health responses, demonstrating extraordinary adaptability and commitment under pressure [8]. This experience has reinforced their critical roles as caregivers, educators, advocates, and community leaders [9,10].

Moreover, the pandemic has highlighted the necessity of holistic healthcare approaches that integrate mental health support, community engagement, and traditional belief systems to enhance public health interventions [11,12]. In Ghana, as in other parts of the world, the intersection of professional practice and cultural context is particularly relevant, as community norms, religious leaders, and traditional healers significantly influence health behaviors and attitudes [13,14,15].

Ghana’s experience during the COVID-19 crisis offers a compelling case study for examining the systemic challenges nurses face. As infection rates surged and routine health services were disrupted, the urgency of rapid, effective health promotion became evident. Nurses not only found themselves in leadership roles but also engaged in public education and provided psychosocial support to both patients and their colleagues. Such experiences align with prior studies’ findings that emphasize resilience-building through leadership in health crises [16,17]. For example, the literature on the Baltimore & Ohio Railroad Museum highlights how organizations can learn and adapt through rare events, revealing both strengths and vulnerabilities [16].

Despite growing recognition of nursing’s critical role in pandemic response, substantial gaps remain in the literature regarding nurses’ experiences in LMICs. Most studies focus on high-resource contexts where health infrastructure and key consumables such as PPEs are not difficult to obtain [18,19,20], leaving the unique challenges and innovations of nurses in Ghana underexplored. This study aims to fill that gap by examining the lived experiences of senior nurses in key health facilities across Ghana during the COVID-19 pandemic. By capturing their narratives, this research aims to illuminate the strengths and the weaknesses of current health systems, identify strategies that foster resilience, and extract lessons that can inform policy and practice. Ultimately, this work contributes to advancing Sustainable Development Goal 3 (SDG 3), which emphasizes the need to ensure healthy lives and promote well-being for all ages [21]. The sustained efforts in health promotion and education during the pandemic provide critical lessons for future pandemic preparedness and for building resilient health systems [22,23].

## 2. Methods

### 2.1. Research Design

This study utilized a qualitative, exploratory, descriptive design to explore senior nurses’ experiences and perspectives on health promotion and education during the COVID-19 pandemic [24]. This approach facilitated the identification of nuanced insights into the challenges nurses faced in their roles during this crisis.

### 2.2. Study Setting

The study was conducted in four regions across nine carefully selected government-funded hospitals designated to manage confirmed COVID-19 cases in Ghana. These regions were among the sixteen regions in the country that trained lead healthcare professionals, including nursing and midwifery managers to also train other healthcare professionals. These hospitals were specifically chosen because senior nurses in these hospitals were among the lead health professionals trained to serve as trainers during the COVID-19 pandemic, and their facilities were among the centers designated to manage cases. Additionally, nurses and midwives are the only healthcare professionals who stay with patients for longer hours and are the most abundant in healthcare facilities. Hence, the centers were selected to focus on the critical roles nurses play in health promotion and education during the pandemic.

The Greater Accra Regional Hospital, the Ga East Municipal Hospital, the University of Ghana Hospital, the University of Ghana Medical Centre, the Tema General Hospital, the Komfo Anokye Teaching Hospital, the Kumasi Government Hospital, the Tamale Teaching Hospital, and the Wa Regional Hospital served as recruitment outlets. These establishments are located in cities with airports where travelers from outside the country land and, therefore, were among the first in the nation to treat both suspected and confirmed COVID-19 cases.

### 2.3. Population, Sampling, and Sample Size

The target population comprised all senior nurses within the selected facilities in the regional capitals that served as epicenters. These senior nurses were from nursing officers and above who, at the beginning of the pandemic, were among those trained to serve as trainers; however, upon Ghana reporting its first positive cases, these trainers, who were working in the regional capitals where cases were found remained the caregivers at the designated treatment centers. Two of the researchers collected data from 30 purposefully selected [25] participants using the snowball sampling technique until they reached saturation [26]. This is a point in data collection where no new theme emerges, and repetitive narrations occur among participants [26]. Saturation in this study occurred across regions where nurses’ narration became repetitive. This method allowed for identifying primary participants who met the inclusion criteria, who then referred the researchers to additional participants. The participant distribution included: fifteen recruited from the Greater Accra Region, five from the Greater Kumasi Region, two from the Northern Region, and three from the Upper East Region. Breakdown by hospital includes: three from the University of Ghana Hospital, four from Greater Accra Regional Hospital, three from the University of Ghana Medical Centre, four from the Ga East Hospital, two from the Komfo Anokye Teaching Hospital, three from the Kumasi Government Hospital, two from the Tamale Teaching Hospital, and three from the Wa Regional Hospital.

### 2.4. Inclusion and Exclusion Criteria

We included all nursing and midwifery managers trained to care for COVID-19 cases and working at the designated COVID-19 isolation and treatment centers. The study did not include other healthcare professionals trained because their involvement as frontline staff was episodic. Also, other nurse managers on leave during the peak of the pandemic at the chosen centers were excluded.

### 2.5. Data Collection Instrument and Procedure

Individual in-depth interviews were conducted from April to May 2020 to obtain rich qualitative data. Semi-structured questions were developed based on study objectives and literature review to ensure the reliability of the information. Some of the questions asked include “How have you promoted health and education regarding the COVID-19 pandemic?” and “What efforts have you made to enhance community resilience during this crisis?” Prior to the main data collection, one researcher conducted a pilot test of the guiding questions with two nurses at one of the centers (the University of Ghana Hospital). The pilot’s outcomes did not alter the guiding questions; however, the researchers learned how difficult it is to recruit frontline staff into the study. The researchers established a good rapport with the participants before commencing the main data collection, ensuring they were informed about the study’s purpose to enhance recruitment. Participants were encouraged to discuss their experiences of health promotion and education during the pandemic in-depth during one-on-one telephone interviews. The interviews lasted between 30 min and one hour.

### 2.6. Data Analysis

Two authors (first and second authors) analyzed the data using Graneheim’s content analysis approach [27], which provided a robust framework for interpreting qualitative data and understanding nuanced human experiences during the COVID-19 pandemic. The researchers first immersed themselves in the interview transcripts, reading and re-reading them to understand the overarching themes from participants’ narratives. The analysis involved a recursive coding process that enabled the researchers to identify meaningful units and assess their significance. After coding, categories were created to organize the data into manageable pieces, encapsulating broader constructs. The research team engaged in collaborative discussions to ensure the reliability and trustworthiness of the themes derived from participant experiences.

To further validate the analysis, three participants voluntarily reviewed the identified themes to confirm that their perspectives were accurately represented. The researchers maintained a detailed record of all coding decisions made throughout the analysis to strengthen transparency and strengthen the credibility of the findings. The final stage involved interpreting the themes, considering the study’s objectives, acknowledging participants’ contributions, while offering actionable insights to enhance community resilience. This was done with the last author who was the P1 of the study.

### 2.7. Rigor

To ensure study trustworthiness and quality, the researchers employed a comprehensive framework described by Guba and Lincoln [28], which emphasizes five key elements: credibility, transferability, dependability, confirmability, and authenticity. Engagement with the data was maintained over time through repeated readings of the interview transcripts to deepen our understanding. Member checking was employed, with four participants reviewing their transcripts to verify that their voices were faithfully captured.

To enhance transferability, the researchers provided rich details about the study setting. This included participants’ demographic characteristics and the specific challenges nurses faced in Ghana during the COVID-19 pandemic. The researchers aimed to provide thick descriptions to allow readers to evaluate the relevance of the findings to other contexts, enhancing their understanding of nurses’ complex realities.

To ensure reliability, the entire research process was systematically documented, capturing decisions made during data collection and analysis. Regular meetings facilitated a collective approach to coding methods and theme development. The researchers practiced reflexivity by maintaining a research diary throughout the process, recording thoughts, biases, and assumptions to examine their potential impacts on the analysis.

Authenticity was enhanced by ensuring a balanced representation of participants’ experiences and by actively including diverse voices from various regions and hospitals. This integrated approach enabled a nuanced understanding of the nursing experience, which is essential for promoting health education and supporting community resilience during unprecedented times.

### 2.8. Ethical Considerations

The study adhered to the principles of ethics outlined in the Helsinki Declaration (1975, revised in 2013) [29]. Ethics approval was granted by the Nursing and Midwifery Council of Ghana’s Ethics Review Committee (IRC No. N&MCIRC/0000002) on 10 March 2020. Data collection began after participants were informed of the study’s purpose and provided informed consent. Participants could withdraw from the study at any time without penalty. To preserve confidentiality, participants’ identities were anonymized using unique codes, and all data were securely stored and encrypted. Considering potential psychological discomfort, a clinical psychologist was arranged to provide mental health support, but no interview required this service.

## 3. Result

The researchers recruited 30 participants for the study. These participants were drawn from various regions and had varied professional backgrounds and experience. After analysis, three main themes and 17 subthemes were identified. Table 1 below shows the themes and subthemes identified. Details of these themes and their subthemes have been presented subsequently, along with their verbatim quotations.

The key findings presented in Table 1 provide a nuanced understanding of how frontline nurses navigated unprecedented challenges, adapted health promotion strategies, and embraced emerging trends to strengthen healthcare resilience.

First, the pandemic revealed both strengths and vulnerabilities within the healthcare system, underscoring nurses’ critical adaptability, teamwork, and leadership amid systemic challenges. Nurses exhibited remarkable resilience by rapidly acquiring new skills, supporting one another, and maintaining their professional identity and unity despite facing increased workloads, psychosocial stress, and limitations in institutional support.

Second, health promotion and education practices emerged as essential pillars for fostering resilient communities. Nurses were proactive in implementing rigorous hygiene, infection prevention, and self-care practices while extending their influence beyond hospital settings to educate and support the broader community. These initiatives, rooted in individual responsibility and systemic support, played a crucial role in sustaining health and morale throughout the crisis.

Lastly, the pandemic expedited technological adaptations and intensified calls for policy advocacy, reshaping communication and collaboration in healthcare settings. Digital platforms and informal telehealth strategies became indispensable tools for continuous learning and safe patient engagement. Concurrently, the crisis galvanized collective demands for improved policies, equitable compensation, and greater recognition of all healthcare contributors.

### 3.1. Theme 1: Nursing Experiences and Systemic Challenges in Pandemic Response

To sustain health promotion and education, it is vital to understand nurses’ lived experiences, especially during crises such as the COVID-19 pandemic. The pandemic imposed extraordinary demands on Ghanaian nurses, shedding light on their resilience and the vulnerabilities within the healthcare system. Nurses were required to adapt swiftly to changing clinical environments, relying on their prior experiences, expedited training, and peer support to bridge knowledge gaps. This collective effort fostered new levels of interprofessional collaboration, as flexible team structures and blurred professional boundaries enabled effective care delivery despite strained conditions.

Despite unwavering dedication, nurses encountered significant challenges, including increased workloads, fatigue, psychosocial stress, and inconsistencies in institutional support. Both internal and community-driven stigma exacerbated these pressures, while systemic flaws and unclear policy directives pointed to areas needing immediate improvement. Nevertheless, these challenges sparked a renewed sense of professional identity, unity, and advocacy among nurses, underscoring the need for stronger leadership, robust policy frameworks, and enhanced investment in staff welfare and mental health. These lessons are critical to sustain health promotion and education, which are foundational for building resilient communities capable of effectively responding to future public health crises.

Through their experiences, Ghanaian nurses highlighted the importance of preparedness, teamwork, transparent communication, and leadership in strengthening frontline resilience and enhancing the broader health system’s capacity to manage crises.

#### 3.1.1. Professional Preparedness and Adaptive Learning

Ensuring professional preparedness and adaptive learning is central to sustaining health promotion and education. During the COVID-19 pandemic, health institutions in Ghana rapidly initiated training programs that incorporated lessons from previous epidemics and integrated online learning to address knowledge gaps. Preparedness was achieved through formal workshops, self-directed learning, and prior clinical experience. Early and peer-led training, complemented by credible online resources, was pivotal in equipping nurses with skills and knowledge to navigate the uncertain disease landscape.

*“We started having workshops, and then I have been reading on my own to get used to it… my experience as a perioperative nurse kind of helped me to understand how to protect myself.”*

(NICA1)

*“So far, this is the first training that I have undergone since this pandemic, that is, this COVID-19 condition.”*

(NICB2)

Nurses adapted to the new disease landscape, drawing parallels with previous infection control protocols while acknowledging the unique risks posed by COVID-19’s unprecedented scale and uncertainty.

#### 3.1.2. Team Structure, Leadership, and Role Flexibility

Building resilient communities during the pandemic depended on interprofessional collaboration and flexible task allocation. Rotating staff through high-risk units and empowering nurse leaders reduced internal stigma and boosted morale. The vital roles of teamwork, multidisciplinary collaboration, and adaptable task allocation were universally acknowledged. Professional boundaries softened, allowing nurses, doctors, and support staff to undertake overlapping responsibilities to minimize exposure and maintain care standards.

*“During the pandemic, staff tasks overlapped. If you are a nurse, you can work as a cleaner; if you are a doctor, you can work as a nurse or a cleaner too.”*

(NICC3)

Leadership emerged both formally and informally, with experienced staff mentoring newcomers and ensuring that all nurses were equipped and prepared for their COVID-19 duties.

#### 3.1.3. Clinical Care, Patient Management, and Communication

Promoting health and education among patients necessitated that nurses provide predominantly symptomatic care, as protocols evolved in real time. Daily routines incorporated vital checks, medication administration, and patient education—essential elements of sustaining health promotion. Effective communication within clinical teams and with patients, particularly in isolation, proved critical for addressing psychosocial needs and preserving empathy despite the barriers posed by PPE.

*“COVID-19… is a virus that is managed symptomatically because currently there is no medication for it, so the condition is managed according to the symptoms the client shows.”*

(NICJI)

Strategies to maintain empathy and humor, despite physical barriers imposed by PPE, were emphasized:

*“They have funny ways of telling us the things they request for and when we come out we laugh about it within ourselves…”*

(NICD2)

#### 3.1.4. Staff Welfare, Fatigue, and Psychosocial Impact

The sustainability of health promotion efforts is closely linked to healthcare staff welfare. While nurses reported high intrinsic motivation and commitment, substantial gaps in welfare support, rest, and incentives were also evident. Fatigue, insomnia, and emotional strain were commonplace issues, exacerbated by prolonged shifts and discomfort from PPE. Institutional support, such as meal provisions, was inconsistently delivered, underscoring the need for policies that safeguard staff well-being as a pillar of resilient communities.

*“It is more of the fatigue, because when I was on night duty, I didn’t sleep…”*

(NICJ1)

The inconsistency in institutional support underscores an urgent need for actionable policies that genuinely care for staff welfare.

#### 3.1.5. Stigma, Community Perceptions, and Coping Mechanisms

The pandemic exposed and amplified stigma from within and outside the healthcare profession. Sustaining health promotion and education required both internal leadership to reduce stigma and community-wide educational efforts to combat fear and discrimination. Nurses utilized various coping strategies, including peer support, humor, faith, and service to family and country, emphasizing the connection between personal resilience and systemic support.

*“The stigma is just too much… even within our health staff.”*

(NICE1)

Targeted mental health interventions and community education are key to mitigating stigma and reinforcing resilience among healthcare workers.

#### 3.1.6. Incident Management, System Lapses, and Policy Gaps

Lessons from the pandemic illustrate that building resilient communities necessitates more than clinical guidelines. Effective incident management relies on robust, clear, and enforceable policies addressing staff welfare, equitable incentives, transparency, and communication. Systemic failures during the pandemic spotlighted vulnerabilities in decision-making and resource allocation, reinforcing the need for comprehensive preparedness strategies.

*“This client came in and was not having the results that he was COVID positive or negative… the medical director said we should put him in the room…”*

(NICB1)

Such challenges highlighted the urgency for well-defined, enforceable policy frameworks.

#### 3.1.7. Professional Identity, Unity, and Leadership Advocacy

The pandemic strengthened nurses’ professional identity and collective advocacy. Unified voices and robust leadership structures are essential to advancing nursing interests and ensuring that frontline perspectives guide system design and resource allocation. Advocacy for health promotion and education is central to sustaining resilience in the face of ongoing and future challenges.

*“Now is the time for us to all rally behind whoever is the head of nursing…”*

(NICD2)

Professional unity and leadership advocacy are critical for engaging with management, ensuring frontline voices shape system developments.

### 3.2. Theme 2: Health Promotion and Education Practices

During the COVID-19 pandemic, building resilient communities hinged on comprehensive health promotion and education strategies adopted by healthcare workers. Staff fostered a culture of rigorous personal and environmental hygiene that became both a duty and a moral commitment. Daily routines included strict hand hygiene, consistent sanitation, and strategic patient interactions as critical safety measures for both staff and patients.

Meticulous PPE use fostered accountability and solidarity, enhancing team cohesion and reinforcing technical safety. Nutrition and physical well-being were prioritized to maintain immunity, supported by both institutional initiatives and personal efforts. In the absence of definitive treatments, healthcare workers implemented proactive measures such as vitamin supplementation and systematic management, informing their strategies through institutional guidelines and peer advice. Their commitment to community outreach extended their impact beyond immediate patient care, ensuring greater public engagement and education.

These practices illustrate the interplay of individual initiative and systemic backing, emphasizing the necessity of personal health practices, resource availability, and ongoing education as key components of resilience and health promotion during public health emergencies.

#### 3.2.1. Personal and Environmental Hygiene Activities

A commitment to rigorous hygiene and infection prevention constituted the backbone of daily routines, with staff viewing these measures as both technical necessities and moral imperatives. Heightened vigilance around hand hygiene and environmental sanitation reflected acute risk awareness, sustained by institutional resources and individual accountability.

*“Preventive measures—you need to be washing your hands…”*

(NIC-A1)

Cleaning protocols extended to all surfaces, signifying an understanding of fomite transmission risks:

*“I have to wash my bowls and everything with chlorine every day…”*

(P)

Staff coordinated access to the wards, limiting entrances to minimize exposure:

*“We limit our contact with the patients such that if today we have to go inside, we plan it…”*

(P)

Ensuring the availability of proper sanitation supplies mirrored institutional support:

*“There’s always liquid soap available with tissues to wash and clean your hands.”*

(H2)

These statements underscore a dual approach of individual responsibility alongside a supportive healthcare system.

#### 3.2.2. Protective Clothing (PPE) and Monitoring

Infection Prevention and Control (IPC) anchors health promotion efforts. PPE use, though challenged by shortages, evolved into a ritualized and peer-monitored process that fortified staff both psychologically and practically. The act of donning PPE became an emblem of group solidarity, essential for maintaining safe care and resilient team dynamics.

*“We were running out of PPEs… we had to start rationing.”*

(NICA2)

Peer accountability and ritualized practices were paramount to preventing lapses:

*“Before we go inside, we put on the PPEs… other colleagues will check to make sure you are well dressed…”*

(NIC-A1)

#### 3.2.3. Nutrition

Nutrition was crucial to pandemic resilience, with supplementation and institutional meal provisions serving as both protective measures and symbols of care. These efforts supported staff immunity and morale, reinforcing the connection between organizational support and health promotion.

*“Every day I take Vitamin C and Zinc…”*

(C3)

Institutional meals, enhanced with nutritional value, symbolized recognition of nurses’ efforts.

*“Breakfast now has added eggs or sausages… to boost their immune system.”*

(NIC-B1)

Nutrition transcended basic sustenance; it represented an organizational acknowledgment that motivated staff.

#### 3.2.4. Physical Activity

Physical activity adapted to the demands of shift work was vital for both physical and mental health. Simple exercises became essential coping strategies and forms of self-care:

*“I walk… I will just pick a trotro (public transport) and then walk to the facility…”*

(C3)

Intentional breathing exercises and light workouts served as effective stress relief:

*“I normally do breathing exercises when I am home… at least some 10 to 20 arm presses…”*

(NIC-A1)

#### 3.2.5. Prophylaxis and Symptomatic Management

Healthcare workers actively engaged in prophylactic and symptomatic care, guided by protocols and peer advice, enhancing their sense of control over an uncertain environment.

*“I am taking… Vitamin C and Zinc… we were told to take it by our facilitators, and we got it from the pharmacy.”*

(C3)

Symptomatic management was standard due to the lack of definitive treatments.

*“COVID-19…I know is a condition…which is being managed symptomatically because currently there is no medication for it…”*

(NIC-A1)

The proactive use of supplements and management of symptoms reflected both institutional guidance and staff’s desire for agency amid uncertainty.

### 3.3. Theme 3: Emerging Trends from the Pandemic

The COVID-19 pandemic accelerated the integration of technology and advocacy in healthcare, setting new precedents for sustaining health promotion and building resilient communities. Digital platforms have become critical for professional communication, education, and coordination, helping bridge information gaps and foster connectedness. Informal telehealth practices emerged, optimizing the use of PPE and minimizing exposure while maintaining patient engagement.

The crisis also intensified calls for clearer medical and social policies, fairer compensation, and broader recognition of all healthcare contributors, including non-clinical staff. Professional advocacy has become more prominent, with frontline workers seeking not only immediate safety but long-term institutional acknowledgment. These emerging trends highlight the importance of technology and advocacy as foundational components in sustaining health promotion and education for resilient communities.

#### 3.3.1. Technology and Virtual Platforms

The pandemic accelerated the adoption of digital communication for professional updates, coordination, and education. Digital platforms became embedded in daily routines, narrowing information gaps and fostering a sense of connectedness. WhatsApp and internet access at work were described as lifelines for information:

*“WhatsApp platforms are being created so if there are updates, they update us through the WhatsApp platform.”*

(NIC-A1)

*“Our buildings do have internet at the nurses’ station, so we go on the internet a lot…”*

(NIC-B1)

Technology was not only a tool but also a new social infrastructure, enabling rapid adaptation and collective learning.

#### 3.3.2. Telehealth and Virtual Medical Strategies

Although formal telehealth was not in place, elements of remote care emerged through phone support and digital information-seeking. These strategies helped optimize PPE use and reduce unnecessary exposure. Remote support for patients via phone reduced physical contact:

*“We have phones… so if they need anything while we have not gone in, they call through the line…”*

(E3)

Staff also utilized online resources for continuous learning:

*“I do go to YouTube, and then I do see videos done by specific doctors… The WHO WhatsApp line, so any updates I just type in, and they give me updates.”*

(NIC-B1)

Digital adaptation was pragmatic, filling the gap left by limited formal telehealth infrastructure.

#### 3.3.3. Medical and Social Policies and Advocacy

The crisis catalyzed advocacy for both clinical and non-clinical staff, with calls for clearer policies, fair compensation, and recognition of all essential workers. Professional associations advocated for safe working conditions:

*“I learned that the GRNMA (Ghana Registered Nurses’ and Midwives’ Association) said without the PPEs we shouldn’t attend to cases…”*

(NIC-A1)

Staff called for equitable recognition and advancement:

*“…if they can promote or jump some of us to the next bid because of this training, especially those at the…if we are promoted, we know that at the end of every month, something small will reflect on our salaries.”*

(C3)

Non-clinical staff’s contributions were highlighted and called out for greater recognition:

*“…the Security… their salaries are low, so if they can be really motivated or given something apart from, we health… they work so hard, they burn the PPEs and washing of boots and a whole lot they work so hard they go into the ward to clean bathrooms, toilets and a whole lot…”*

(E3)

Advocacy and collective bargaining became more prominent, with frontline workers seeking not only immediate safety but long-term structural recognition and fair treatment for all contributors.

## 4. Discussion

The findings from this study offer nuanced insights into frontline nurses’ experiences during the COVID-19 pandemic in Ghana. These findings emphasized the pivotal role of health promotion, education, and emerging trends in building resilient communities. The themes that were generated resonate with broader literature, revealing both congruence and divergence with prior research on pandemic responses, nursing resilience, and systemic supports [30]. Moving forward, these insights should inform targeted interventions and policy reforms to strengthen the nursing workforce and healthcare systems in Ghana and similar settings.

Reflecting on the rapid adaptation and professional preparedness demonstrated by nurses, these align with global reports of healthcare workers’ agility during COVID-19 [31]. Nurses in Ghana, akin to those in other low- and middle-income countries, relied heavily on peer-led learning and rapid, often informal, knowledge transfer, an adaptive strategy also documented by Shanafelt et al. [32]. However, unlike in some high-resource settings where formalized training and preparedness drills were more prevalent [33], Ghanaian nurses faced significant resource constraints, underscoring the importance of context-specific adaptive learning. To enhance future preparedness, it is recommended that health institutions in resource-constrained settings develop structured in-service and online training modules that can be rapidly deployed during crises. Investment in simulation-based preparedness and regular refresher training will further build adaptive capacity among frontline workers.

Teamwork, flexible leadership, and role overlap were critical to sustaining care under pressure. This echoes findings by Fernandez et al. [32], who stressed the value of multidisciplinary collaboration in crisis situations. The blurring of professional boundaries, while effective for rapid response, sometimes led to role ambiguity and increased stress, as noted in the recent literature [33]. These dynamics underscore the need for clear protocols and supportive leadership to harness the benefits of flexible team structures while mitigating potential negatives. Going forward, healthcare managers should institutionalize regular team debriefings and clarify role expectations during emergencies, ensuring that flexibility does not come at the expense of staff well-being or patient safety.

Staff welfare also emerged as both a facilitator and barrier to resilience. While intrinsic motivation and peer support thrived, psychosocial stress, inconsistent institutional support, fatigue, and perceived broken promises regarding incentives undermined morale, a finding supported by Afulani et al. [34] and Shaukat et al. [33]. Studies from both the Ebola and COVID-19 responses in West Africa similarly underscore the toll of inadequate welfare and highlight the critical role of staff well-being in sustaining health promotion efforts [34]. Policymakers should prioritize comprehensive staff welfare packages, including mental health support, timely incentives, and adequate rest periods as core components of emergency preparedness and response strategies. Establishing transparent communication channels and fulfilling institutional promises will foster greater trust and morale among frontline staff.

The persistence of stigma, both internal and community-driven, remains a formidable challenge, consistent with prior epidemics [35,36]. Effective strategies to counteract stigma, such as leadership-driven rotation and community education, are echoed in the literature as essential for enabling resilient health systems [37]. To tackle stigma, health authorities should implement ongoing anti-stigma campaigns, provide training in psychosocial support for healthcare workers, and foster community engagement initiatives that humanize frontline workers and dispel misinformation. Systemic lapses and policy gaps, particularly relating to incident management and resource allocation, mirror concerns documented globally [28,38]. Transparent policies and clear communication are identified in the literature as preconditions for effective pandemic response [37,39]. As a way forward, it is imperative to conduct systematic policy reviews after each health crisis, involving direct input from frontline staff. Developing robust, enforceable protocols and ensuring accountability in resource allocation will fortify system resilience.

The emphasis on personal and environmental hygiene, PPE adherence, nutrition, and physical activity corroborates WHO guidelines and best practices outlined in infection prevention literature [40]. Peer monitoring and ritualized PPE routines, as found in this study, have been shown to enhance compliance and solidarity [41]. However, resource shortages and anxiety over supply chains present ongoing challenges, as documented in both local and international contexts [31,42,43,44]. Healthcare administrators should ensure sustained investment in supply chain management for essential infection-prevention supplies and promote a culture of safety and peer accountability. Periodic audits and drills can help maintain high standards and quickly address gaps as they arise. Nutrition and physical activity as self-care strategies further underscore the importance of holistic health promotion in crisis settings. The literature supports the role of adequate nutrition and physical resilience in mitigating stress and supporting immunity [45,46]. Institutional support, such as meal provision, has been shown to boost morale and perceived organizational support [36]. Health institutions should integrate wellness programs, including nutritional support, exercise opportunities, and counseling into their staff welfare initiatives, both in emergency and routine contexts.

Nurses’ roles as community educators and sources of up-to-date information highlight the profession’s evolving scope during pandemics [37]. This dual function, educating both patients and the wider community, has been recognized as central to effective public health response [41]. However, the study also reveals gaps in formal community outreach infrastructure, consistent with findings from other resource-constrained settings [38]. To advance health promotion, partnerships should be forged between healthcare facilities, local governments, and civil society to strengthen community outreach and education, especially in rural and underserved areas. Continuous professional development in health communication should be prioritized for all nurses.

The accelerated adoption of digital platforms for communication, education, and informal telehealth is consistent with global trends [47,48]. In Ghana, the pragmatic use of WhatsApp and other online resources filled gaps left by limited formal infrastructure, supporting findings by Afulani et al. [35] that digital adaptation can bridge information and coordination gaps in low-resource contexts [49,50]. Efforts should focus on strengthening healthcare’s digital infrastructure, investing in secure, scalable telehealth platforms, and providing digital literacy training for all staff [51,52]. This will enhance preparedness and continuity of care in future health emergencies.

Professional advocacy and calls for policy reform are aligned with global movements for improved recognition and welfare for healthcare workers [31,40]. The study findings underscore the importance of unified professional identity and collective bargaining, as highlighted in the literature, to ensure that frontline perspectives inform system design and resource allocation [36,42]. Nursing associations and other professional bodies should be empowered and supported to advocate for equitable policies, fair compensation, and recognition for all healthcare contributors, including non-clinical staff. Mechanisms for regular dialogue between policymakers and frontline workers should be established to ensure responsive and inclusive decision-making.

## 5. Limitations of the Findings

While the findings align with much of the existing literature, there are several notable contrasts and limitations that warrant discussion. For instance, in higher-income contexts, formal telehealth and remote care infrastructures were rapidly scaled up, providing structured frameworks for patient engagement and care continuity [48]. In contrast, Ghana primarily relied on informal and ad hoc solutions that may lack the efficacy and integration found in more developed systems.

Additionally, the variability in institutional support observed in this study contrasts sharply with reports from some countries where staff welfare interventions were more robust and consistent [33]. This discrepancy highlights the need for systemic reforms in Ghana to foster a more supportive environment for healthcare workers.

A critical methodological limitation pertains to the use of telephone interviews for data collection. While this mode enabled broader participation and facilitated timely data collection, it also introduced potential challenges. For instance, the lack of face-to-face interaction affected the depth of responses and the ability to gauge nonverbal cues, both crucial for understanding nuanced emotions and expressions during discussions. To mitigate this limitation, researchers employed active listening techniques and followed up on key points to clarify, ensuring rich, meaningful data collection despite the constraints of distance. Furthermore, reliance on informal practices may lead to inconsistencies in care quality and access to resources, a concern less prevalent in higher-resource settings. These limitations suggest that, while valuable insights can be drawn from Ghanaian nurses’ experiences, contextual factors and methodological considerations influence the effectiveness of health promotion and education strategies.

To bridge these gaps, international collaborations and knowledge exchange programs are critical. Such initiatives could facilitate the adaptation of best practices from high-resource settings, ensuring they are tailored to fit local realities. Policymakers in Ghana should prioritize investments in building institutional capacity for both digital health infrastructure and comprehensive support systems for healthcare workers. By addressing these limitations, the healthcare system can enhance resilience and better respond to future public health challenges.

## 6. Key Contribution

- Insights into Frontline Experiences: The study offers valuable insights into the unique experiences and challenges that frontline nurses in Ghana faced during the COVID-19 pandemic.
- Contextualized Findings: It highlights differences and similarities in nursing responses across high-income countries and other low- and middle-income contexts, contributing to a more nuanced understanding of health system resilience.
- Recommendations for Policy and Practice: The findings offer actionable recommendations to strengthen nursing practice and enhance emergency preparedness in resource-constrained settings.
- Framework for Future Research: The study lays the foundation for future investigations into pandemic responses among healthcare professionals in low- and middle-income countries.

## 7. Conclusions

Overall, this study reinforces that sustaining health promotion and education in crisis contexts is a multidimensional challenge requiring both individual agency and systemic support. Lessons learned from Ghanaian nurses’ experiences during COVID-19 offer unique perspectives on health system resilience, staff welfare, and the centrality of frontline workers in driving community health outcomes. A concerted effort involving policy reform, investment in staff welfare, digital innovation, and community engagement will be pivotal to building truly resilient healthcare systems capable of withstanding future public health emergencies.

## Figures and Tables

**Table 1:** Themes and subthemes.

Themes/Subthemes
1. Nursing Experiences and Systemic Challenges in Pandemic Response (a) Professional Preparedness and Adaptive Learning (b) Team Structure, Leadership, and Role Flexibility (c) Clinical Care, Patient Management, and Communication (d) Staff Welfare, Fatigue, and Psychosocial Impact (e) Stigma, Community Perceptions, and Coping Mechanisms (f) Incident Management, System Lapses, and Policy Gaps (g) Professional Identity, Unity, and Leadership Advocacy
2. Health Promotion and Education Practices (a) Personal and Environmental Hygiene Activities (b) Protective Clothing (PPE) and Monitoring (c) Nutrition (d) Physical Activity (e) Prophylaxis and Symptomatic Management (f) Community Outreach and Education
3. Emerging Trends from the Pandemic (a) Technology and Virtual Platforms (b) Telehealth and Virtual Medical Strategies (c) Medical and Social Policies and Advocacy

## Data Availability

The data from which this paper was developed is available on reasonable request from the corresponding author.
